# A Closer Look at the Bivariate Association between Ambient Air Pollution and Allergic Diseases: The Role of Spatial Analysis

**DOI:** 10.3390/ijerph15081625

**Published:** 2018-08-01

**Authors:** Dohyeong Kim, SungChul Seo, Soojin Min, Zachary Simoni, Seunghyun Kim, Myoungkon Kim

**Affiliations:** 1 School of Economic, Political and Policy Sciences, The University of Texas at Dallas, 800 W Campbell Road, Richardson, TX 75080, USA; dohyeong.kim@utdallas.edu (D.K.); soojin.min@utdallas.edu (S.M.); zrs160030@utdallas.edu (Z.S.); 2 Department of Environmental Health and Safety, College of Health Industry, Eulji University, 553 Sanseong-Daero, Sujeong-Gu, Seongnam-Si, Gyeonggi-Do 13135, Korea; 3 Department of Medical Biochemistry & Molecular Biology, Korea University, Anam-dong, Seongbuk-gu, Seoul 136-701, Korea; seunghyun@korea.ac.kr (S.K.); jerrykim@korea.ac.kr (M.K.)

**Keywords:** allergic disease, air pollution, bivariate association, Geographic Information Systems, spatial analysis

## Abstract

Although previous ecological studies investigating the association between air pollution and allergic diseases accounted for temporal or seasonal relationships, few studies address spatial non-stationarity or autocorrelation explicitly. Our objective was to examine bivariate correlation between outdoor air pollutants and the prevalence of allergic diseases, highlighting the limitation of a non-spatial correlation measure, and suggesting an alternative to address spatial autocorrelation. The 5-year prevalence data (2011–2015) of allergic rhinitis, atopic dermatitis, and asthma were integrated with the measures of four major air pollutants (SO_2_, NO_2_, CO, and PM_10_) for each of the 423 sub-districts of Seoul. Lee’s L statistics, which captures how much bivariate associations are spatially clustered, was calculated and compared with Pearson’s correlation coefficient for each pair of the air pollutants and allergic diseases. A series of maps showing spatiotemporal patterns of allergic diseases at the sub-district level reveals a substantial degree of spatial heterogeneity. A high spatial autocorrelation was observed for all pollutants and diseases, leading to significant dissimilarities between the two bivariate association measures. The local *L* statistics identifies the areas where a specific air pollutant is considered to be contributing to a type of allergic disease. This study suggests that a bivariate correlation measure between air pollutants and allergic diseases should capture spatially-clustered phenomenon of the association, and detect the local instability in their relationships. It highlights the role of spatial analysis in investigating the contribution of the local-level spatiotemporal dynamics of air pollution to trends and the distribution of allergic diseases.

## 1. Introduction

Although numerous studies report that genetic factors play a critical role in determining susceptibility or exacerbation of allergic diseases such as atopic dermatitis, asthma, and allergic rhinitis [1], recent work has been dedicated to environmental exposure to better understand the prevalence of allergic diseases, since a significant change in human genetics could not occur in such a short period [2]. The increased prevalence of allergic diseases has been found in “westernized” or “modernized” countries such as South Korea, and this epidemiologic change has been attributed to the augmented level of air pollution in those countries [3]. Most of the research investigates ambient air pollutants, including particle matters (PM_10_ and PM_2.5_), nitrogen dioxide (NO_2_), carbon monoxide (CO), sulfur dioxide (SO_2_) and ozone (O_3_) [4]. Although the majority of studies investigating the relationship between air pollutants and allergic diseases have been cross-sectional, observational, or longitudinal cohort studies, there are relatively few ecological studies [5] due to susceptibility to the ecological fallacy. The ecological fallacy refers to the possibility of making misleading inferences based on grouped individuals [6]. Moreover, as areal data is often used in spatial analysis, the ecological fallacy may lead to the Modifiable Areal Unit Problem (MAUP), which produces widely varied analytical results depending on the level of spatial aggregation of data [7]. The MAUP effect causes the inference to change depending on the size of aggregated geographical areas or the rules of forming areal units [8]. One study found that regional and local variations in asthma prevalence were not observed by national level data, since aggregation of data masks significant spatial variations in the outcomes [9]. Thus, using fine geographic granularity is recommended to adequately address the MAUP effect [10].

Although the MAUP effect is essentially a matter for all quantitative studies involving areal data [11], ecological research can be beneficial in a number of ways. First, readily available, timely data, often used by ecological research, allows low resource-intensive research. Due to this advantage, the ecological approach has been widely used in various environmental health studies [12,13,14,15]. It is also the case in research on air pollution related allergic diseases. Hospitals generally maintain electronic health records such as numbers of patient visits or admissions, whereas governments manage and release air pollution statistics on a regular basis. Some studies have used routinely-collected health records to explore the ecological connection between air pollution and allergic diseases which may have been neglected by previous individual-level studies [16,17]. Moreover, the ecological approach helps understand the intricate health effects of outdoor air pollution by comparing health outcomes in populations across gradient levels of air pollutants [18].

Although previous ecological studies investigating the association between air pollution and allergic diseases accounted for temporal or seasonal relationships [16,19,20,21], few studies consider spatial aspects in their analyses, including spatial autocorrelations. Ecological variables in geographically referenced data sets tend to be dependent from one another within a relatively close range due to spatial autocorrelation [22]. The information from spatially auto-correlated observations is different from independent observations, as a certain amount of the information from each observation is duplicated within the cluster [23]. Thus, because the presence of spatial autocorrelation is often observed in environment or health related variables [24,25], it must be addressed when studying the ecological association between air pollution and allergic diseases. Failure to do so will increase the likelihood of inaccurate statistical decisions such as Type I errors [22]. Therefore, addressing the nature of spatial heterogeneity of both environmental and health variables helps interpret the “true” effect between air pollutants and prevalence of allergic diseases. Due to these factors, spatial perspectives are vital for an adequate interpretation of ecological analyses using aggregated areal data. Research on air pollution-related allergic diseases needs to acknowledge such properties intrinsic to spatial data representing geographical variations of the prevalence of allergic diseases and levels of outdoor air pollutants. While only a few studies consider univariate or bivariate spatial autocorrelation when estimating the impact of air pollution on allergic diseases [26,27], no study has accounted for a combinational effect of spatial autocorrelation of each measure and exposure-to-disease correlation between air pollutants and the disease prevalence.

In South Korea, spatial analysis of allergic disease related research involving ambient air pollution is lacking. Only one recent study considered spatial non-stationarity in researching the association between allergic diseases and air pollutants, but ignored spatial autocorrelation [17]. Recent studies based on national level data reported that the prevalence of asthma and atopic dermatitis demonstrated decreasing prevalence in South Korea [28,29], but these findings could be due to the MAUP effect, where spatially varied prevalence at the finer levels of geography may have been masked by national level observation. There is no indication that the levels of ambient air pollutants have been substantially decreasing in South Korea [30], so unless a significant and steady decrease in the associated risk factors were present, this trend may be a temporary phenomenon. Although levels of air pollutants including SO_2_, NO_2_, and PM_10_ in major cities have not considerably increased in the last 10 years [31], air pollution in Seoul, particularly PM_10_, has increased more compared to other large cities such as Paris, Los Angeles, and London [32,33]. A small number of studies on allergic diseases conducted in Korea have taken into account spatial autocorrelation in ambient air pollutants [34,35], while others focus upon the prevalence of allergic diseases [36]. However, no studies simultaneously account for spatial autocorrelation in their bivariate analysis.

Thus, this study examines 5-year prevalence trends (2011–2015) of three allergic diseases—allergic rhinitis, atopic dermatitis, and asthma—in terms of regional differences, highlights several methodical limitations in the literature, and suggests an alternative to address them. It first investigates spatial autocorrelation in four major air pollutants (SO_2_, NO_2_, CO and PM_10_) and the prevalence of allergic diseases among 423 sub-districts in Seoul. Next, bivariate correlation between each air pollutant and allergic disease prevalence is examined by Pearson’s correlation coefficients and its alternative measure, which are compared to each other in a cross-sectional manner and its trend over time. Lastly, this study assesses the role of spatial analysis, and provides policy implications in terms of how to design public health policies in order to efficiently address the gravity of the prevalence of allergic diseases in South Korea.

## 2. Materials and Methods

Emergency room visits or admission counts have been used as an outcome measure of disease exacerbation, but mostly for severe or acute asthma, rather than for atopic dermatitis or allergic rhinitis [37,38]. In other words, those metrics are permissible for assessing the risk of short-term exposures to air pollutants, but are limited at explaining the long-term effects [39]. Allergic diseases are related to immune disorders attributed to genetic and environmental factors [40]; children with immature immune systems would be relatively more vulnerable to allergic diseases [41]. Thus, we used patient-count data only for children under 12 in order to investigate the long-term effect of air pollution exposure in early childhood. By using data collected by the National Health Insurance Corporation (NHIC) of South Korea from 2011 to 2015, we calculated the number of patients per 10,000 children under 12 for allergic rhinitis, atopic dermatitis, and asthma for each of the 423 sub-districts of Seoul. We chose sub-district as the unit of analysis because it is the most disaggregated administrative unit at which the patient count data is currently available in Seoul. A sub-district is the smallest level of urban government to have its own office and administrative staff in South Korea, and hence, many administrative and public data are collected at this bureaucratic level.

The classification of three types of allergic diseases was based on the disease codes defined by the 11th revision of the International Classification of Diseases (ICD-11): J30 for vasomotor and allergic rhinitis, L20 for atopic dermatitis, and J45-46 for asthma and status asthmaticus. Each patient was not counted more than once, even if the patient had received multiple treatments under the same disease code. We created a series of maps at the sub-district level for each year to examine whether spatial and temporal variations in the prevalence of each allergic disease were observable across the units. We found no significant difference in these data between male and female, so they were analyzed together in all subsequent analyses.

**Figure 1:**
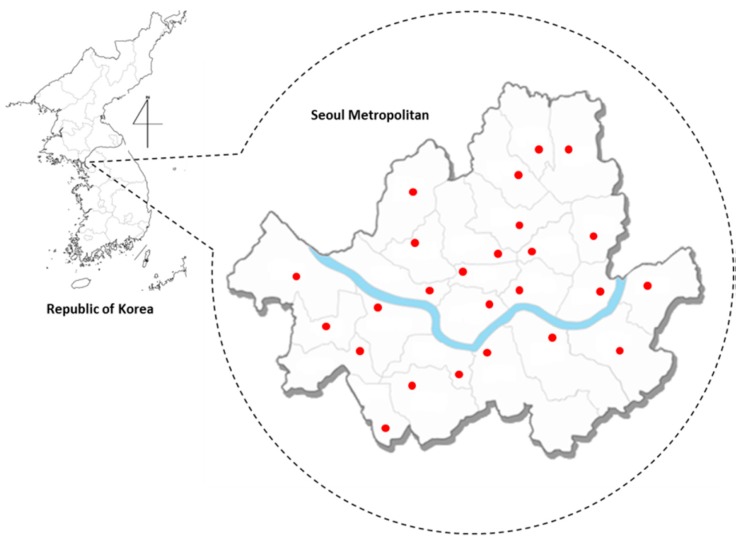
The distribution map of 25 monitoring stations for air pollutants (●), one in each administrative district of Seoul.

The ambient air pollution data collected by the Ministry of the Environment of Korea included daily measurements of four major air pollutants—SO_2_ (ppb), NO_2_ (ppb), CO (ppb), and PM_10_ (μg/m^3^)—at 25 monitoring stations in Seoul (Figure 1). SO_2_, NO_2_, and CO are measured every five minutes, and PM_10_ is monitored each hour. Then, the 1-h mean concentrations of all pollutants are recorded and uploaded to the data archive system. The sampling methods of each pollutant are as follows: SO_2_ the pulse U.V. fluorescence method; NO_2_ the chemilum inescent method; CO the non-dispersive infrared method; PM_10_ for β-ray absorption method. We examined how the concentration levels of each pollutant in Seoul have changed over time for the study period (2011–2015). As there is only one monitoring station for air pollution in each district of Seoul, it is very unrealistic and misleading to assume that the measurement at the monitoring station is the same within the district [42]. So, we estimated the annual average concentration level of each air pollutant for each year and for each sub-district to match the spatial scale of the patient count data using the ordinary kriging method, which has been widely used as a spatial interpolation tool for air pollution measures in the literature [17,43]. We applied the spherical model to the log-transformed data for interpolation, as its assumption tends to be consistent with the spatial distribution pattern of ambient air pollutants [44].

We first checked if spatial heterogeneity in our main variables were consistent over the five years, and found the spatial patterns remained consistent over time. We were thus able to look into spatial and temporal heterogeneity separately. A univariate spatial autocorrelation was estimated for each allergic disease prevalence for each year and the corresponding air pollutant using a global Moran’s coefficient, in order to evaluate whether or not there is a considerable degree of spatial autocorrelation in both data. With the interpolated measurements of the four pollutants in each sub-district, a series of bivariate correlation analyses were performed to scrutinize ecological associations between each ambient air pollutant and the prevalence of allergic diseases at the sub-district level in Seoul. Furthermore, we investigated how the associations changed over time. One of the classical methods of measuring a bivariate association is the Pearson’s correlation coefficient, which can be written for variables *X* and *Y* as follows:

$$r_{X,\;Y}=\frac{\sum_{i}\left(x_{i}-\bar{x}\right)\left(y_{i}-\bar{y}\right)}{\sqrt{\sum_{i}{\left(x_{i}-\bar{x}\right)}^{2}}\cdot \sqrt{\sum_{i}{\left(y_{i}-\bar{y}\right)}^{2}}}$$

However, aspatial association measures, including Pearson’s correlation coefficient, do not recognize the spatial phenomenon of data sets [23]. Thus, these measures face a serious shortcoming for hypothesis testing and prediction when bivariate associations are spatially clustered [45]. Although the bivariate spatial autocorrelation measures based on Wartenberg’s original framework of multivariate spatial correlation (i.e., bivariate global and local Moran’s I) have been widely used to examine the exposure-health relationship in various environmental health studies [26,46,47] via the GeoDa package [48], they fail to incorporate spatial smoothing scalers of both *X* and *Y* variables. Rather, they can only gauge the relationship between a variable and the other variable’s spatial lag [49].

As a better alternative for overcoming such limitations, Lee’s L statistic for bivariate spatial association captures the spatial co-patterning by integrating a univariate spatial autocorrelation of each variable and their bivariate point-to-point associations [49]. Let $\tilde{x_{i}}=\sum_{j}w_{ij}x_{j}$ and $\tilde{y_{i}}=\sum_{j}w_{ij}y_{j}$ where $w_{ij}$ is an element of a row-standardized spatial weight matrix W, and $r_{\tilde{X},\tilde{Y}}$ be a Pearson’s R between the spatial lag vectors of *X* and *Y*. Then, *L* statistics can be written as:

$$L_{X,Y}=\sqrt{\frac{\sum_{i}{\left(\tilde{x_{i}}-\bar{x}\right)}^{2}}{\sum_{i}{\left(x_{i}-\bar{x}\right)}^{2}}}\cdot r_{\tilde{X},\tilde{Y}}=\sqrt{\frac{\sum_{i}{\left(\tilde{x_{i}}-\bar{x}\right)}^{2}}{\sum_{i}{\left(x_{i}-\bar{x}\right)}^{2}}}\cdot \sqrt{\frac{\sum_{i}{\left(\tilde{y_{i}}-\bar{y}\right)}^{2}}{\sum_{i}{\left(y_{i}-\bar{y}\right)}^{2}}}\cdot \frac{\sum_{i}\left(\tilde{x_{i}}-\bar{\tilde{x}}\right)\left(\tilde{y_{i}}-\bar{\tilde{y}}\right)}{\sqrt{\sum_{i}{\left(\tilde{x_{i}}-\bar{\tilde{x}}\right)}^{2}}\cdot \sqrt{\sum_{i}{\left(\tilde{y_{i}}-\bar{\tilde{y}}\right)}^{2}}}$$

As seen in the equation, the *L* statistic is defined as an adjusted Pearson’s R between variables’ spatial lags scaled by the square root of the bivariate spatial smoothing scalar. For environment and health data which tend to be spatially correlated, it can function as a superior alternative to Pearson’s correlation coefficient, as it captures how much bivariate associations are spatially clustered. Likewise, the local measure of *L* statistics ($L_{i}$) can help investigate a bivariate spatial heterogeneity by detecting the local instability in relationships between the two variables *X* and *Y*, which is presented as follows:

$$L_{i}=\frac{n\cdot \left(\tilde{x_{i}}-\bar{x}\right)\left(\tilde{y_{i}}-\bar{y}\right)}{\sqrt{\sum_{i}{\left(\tilde{x_{i}}-\bar{x}\right)}^{2}}\cdot \sqrt{\sum_{i}{\left(\tilde{y_{i}}-\bar{y}\right)}^{2}}}$$

In this study, both Lee’s L and Pearson’s R statistics were estimated and compared for each pair of air pollutants and allergic disease prevalence. The map of the local version of *L* statistics for bivariate association of each pair was also created to demonstrate local patterns of spatial correlation between air pollutant concentration and the number of allergic disease patients, highlighting sub-regions into the four categories such as high-high and low-low locations (spatial clusters) and high-low and low-high locations (spatial outliers).

## 3. Results

**Figure 2:**
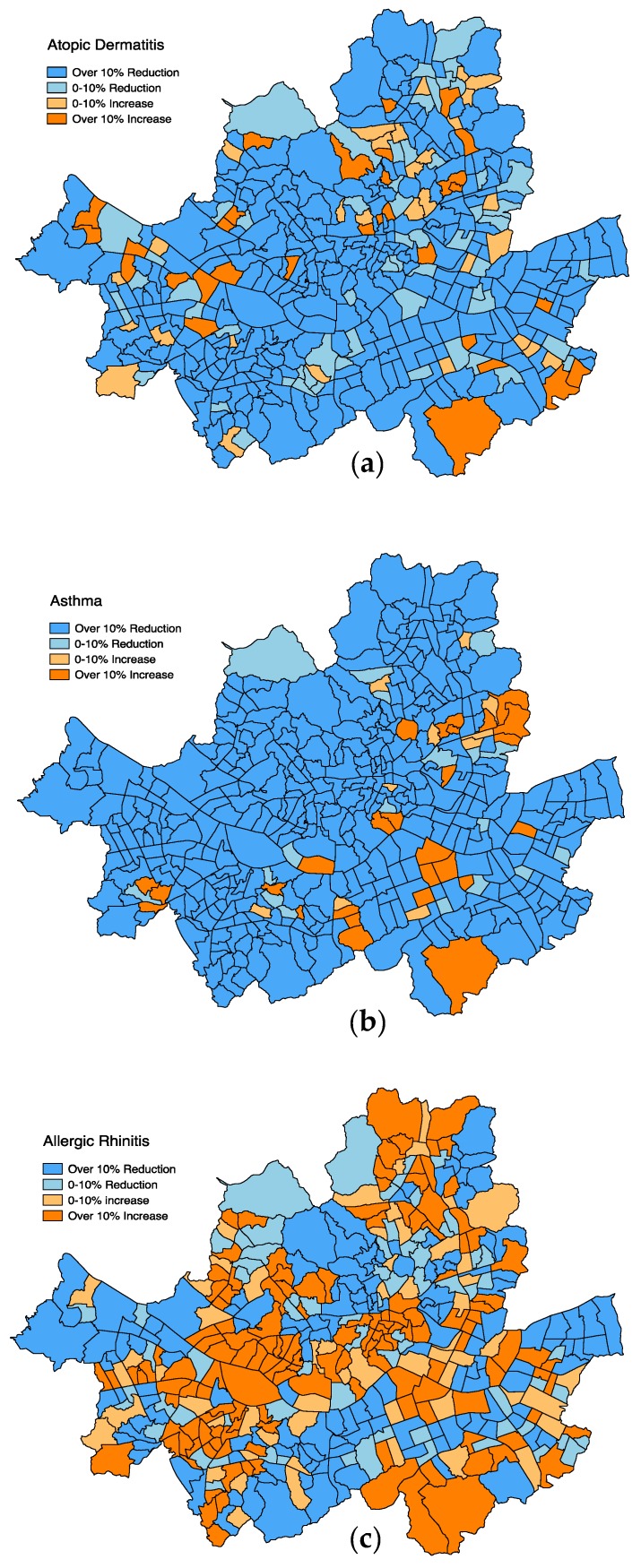
Changes in allergic disease prevalence at 423 sub-districts in Seoul (2011–2015): (**a**) atopic dermatitis; (**b**) asthma; (**c**) allergic rhinitis.

A series of spatiotemporal maps for allergic disease patient counts in Seoul confirmed that our findings followed the national trend of decreasing prevalence of atopic dermatitis and asthma and the increasing prevalence of allergic rhinitis in South Korea [28,29]. However, as shown in Figure 2, the 4-year changes in spatiotemporal patterns for each allergic disease at the sub-district level in Seoul between 2011 and 2015 indicate that some sub-districts in Seoul do not follow the national patterns. Although the prevalence of allergic rhinitis and asthma has decreased in most of the sub-districts in Seoul since 2011, it remains similar or even increased in 10–20% of the sub-districts. Only about 5% of the sub-districts were found to be regions where prevalence increased for all three diseases, which were dispersed throughout the city. Such spatial heterogeneity is even greater on allergic rhinitis. Roughly half of the sub-districts show decreasing patterns, while the other half display increasing patterns. Such differentials for trends of allergic disease prevalence highlight the limitations of previous study findings based on national aggregates because the spatially varied prevalence of allergic diseases at the finer levels of geography may have been masked by national level observation.

**Figure 3:**
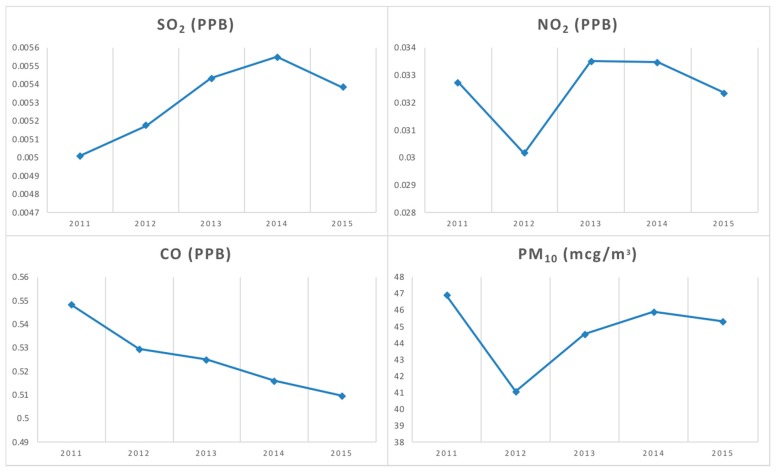
Changes in ambient air pollutants in Seoul between 2011 and 2015.

In addition, unless a significant and steady decrease in the associated risk factors was present, a decreasing prevalence trend could be a temporary phenomenon. Figure 3 shows a temporal pattern of yearly average concentrations for the four major ambient pollutants. All pollutants except carbon monoxide (CO) appear to be either steady or even increasing between 2011 and 2015 in Seoul. However, this kind of report could be misleading due to its aggregated process. This finding emphasizes the need for research efforts to explore the detailed spatial patterns of disease prevalence, as well as its underlying risk factors, in order to better interpret the temporal trends of allergic diseases. This study found substantial spatial heterogeneity in all four air pollutants, and that the patterns remained consistent over the five years. Maps of all four air pollutants by sub-district are available upon request from the authors.

One of the intrinsic spatial aspects characterizing both ambient air pollutants and allergic diseases is spatial autocorrelation. Table 1 confirms a strong degree of spatial autocorrelation for each air pollutant and allergic disease, measured by a global Moran’s coefficient ranging from 0.25–0.39 for allergic diseases and 0.71–0.98 for air pollutants. Such a high level of spatial autocorrelation could make an aspatial bivariate association measure such as Pearson’s correlation coefficient biased and inefficient, since it fails to address spatially-clustered phenomenon of the association.

Table 2 compares the Pearson’s R and Lee’s Global L measures for every pair between the four ambient air pollutants and the three allergic disease patient counts (only children under 12) during 2011 and 2015. The major findings from this table include: (1) for some pairs, the Lee’s Global L is found to be significant with a correct sign (positive) while the Pearson’s R is not significant; (2) for the pairs where the Pearson’s R has a negative sign (which is counter-intuitive), the Lee’s Global L becomes insignificant; (3) for the pairs where both measures are statistically significant at 5% level, the Lee’s Global L tends to be smaller than the Pearson’s R. Despite some fluctuation over time, this finding confirms the previous literature suggesting the significant bivariate association between atopic dermatitis and PM_10_ [17,50]. Although the practical interpretation is limited due to the bivariate structure of the model, we argue that this evidence is sufficient to demonstrate that Lee’s L measure could work as a superior alternative to Pearson’s correlation coefficient, because the presence of spatial autocorrelation in the data may alter the parameter estimates and error probabilities of the metrics.

**Figure 4:**
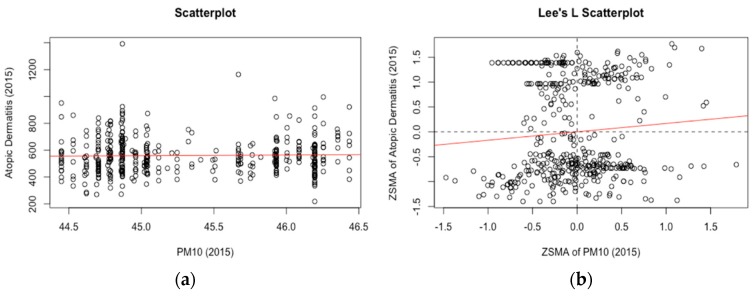
Scatterplots with (**a**) observed points and (**b**) Lee’s L scatterplot with Z-transformed spatial moving average illustrating association between PM_10_ and atopic dermatitis (2015).

Figure 4 illustrates the case where the Lee’s L becomes significant, while the Pearson’s R shows no association. The classical scatterplot shows a one-to-one association between the crude measure of PM_10_ and atopic dermatitis patient counts (children under 12) over 423 sub-districts in Seoul during 2015. On the other hand, Lee’s L scatterplot matches the Z-transformed spatial moving average for each of the same pair, which combine a univariate spatial autocorrelation of each variable with their bivariate point-to-point associations. The slope of the red line in the Lee’s scatterplot indicates the L measure (0.045). Although it is small, the positive association between PM_10_ and atopic dermatitis could have been masked if examined by a classical bivariate association measure ignoring their spatial co-patterning.

**Figure 5:**
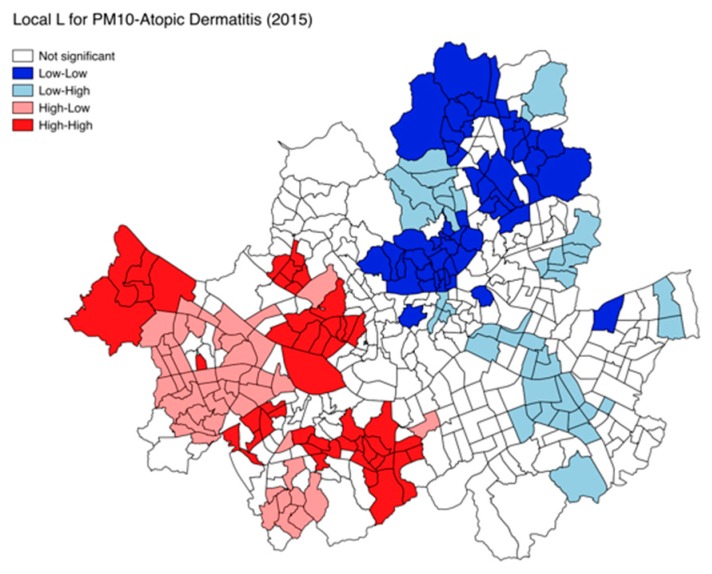
Map of Lee’s Local L for bivariate association between PM_10_ and atopic dermatitis in Seoul.

Despite some annual fluctuation and variation by age group, the Lee’s L measures identify ozone as a major risk factor for asthma and allergic rhinitis, but SO_2_, CO, and PM_10_ as core determinants for atopic dermatitis, during the past five years. However, such finding is also based on a global measure, and the local association patterns could vary by location. Similar to the Anselin’s local indicators of spatial association (LISA), the Lee’s L statistics can be calculated as a local-level indicator and illustrated as a map highlighting “hotspots” and “coldspots”. “Hot spots” refer to a clustering of high air pollutant concentrations and clustering of high allergic disease prevalence, whereas “cold spots” refer to the clustering of low air pollutant concentrations and clustering of low allergic disease prevalence. Figure 5 demonstrates that there are several hot and cold spots for bivariate association between PM_10_ and atopic dermatitis (2015) in Seoul, along with other areas displaying negative associations between them (high-low and low-high). While much effort should be given to explaining the reason behind such local patterns, it would at least identify the areas where a specific air pollutant is considered to be contributing to a type of allergic disease, and thus, should be targeted for regional-specific intervention.

## 4. Discussion

The evidence from this study suggests that evaluating disease trends based on aggregated data may be misleading. Thus, it is crucial to include local-level spatiotemporal disease patterns to more effectively design environmental health policy. This argument is even more potent for allergic diseases, where various risk factors typically show substantial spatial and temporal variations. The driving factors for spatiotemporal variation in allergic diseases at different scales remain unknown and controversial, but this study highlights the role of air pollution in explaining the prevalence and severity of allergic diseases, and emphasizes the importance of taking spatial dynamics, particularly spatial autocorrelation, into careful consideration when measuring their ecological association. The findings in this study support previous ecological research by the authors that geographical variations in prevalence of allergic diseases may be explained by varying levels of air pollutants over space, rather than by individual risk factors [17]. This study also notes that recent decreasing trends of atopic dermatitis and asthma in South Korea reported in the literature may be artifacts of aggregation [28]. As such, they should be re-examined at a more granular level, particularly considering that most of the ambient pollutant concentrations have been either steady or even increasing recently in many areas.

Spatial data disaggregation is imperative to identifying regional-specific or area-specific risk factors, because it would be the first step to derive and establish a cost-effective targeted intervention in reducing the burden of allergic diseases. The comprehensive spatiotemporal patterns of allergic diseases at different scales in Korea presented in this paper could potentially be explained by further multivariate studies exploring the spatially-varying impacts of multiple indoor and outdoor risk factors if the data were available at more disaggregated spatial and temporal scales. Despite recent popularity in the use of Geographic Information Systems (GIS) in visualizing and analyzing spatial patterns for allergic diseases in South Korea [17], most data on disease prevalence, as well as environmental, meteorological, and socioeconomic risk factors, are still typically maintained at highly aggregated levels. For more widespread spatiotemporal environmental health research, the routine public health and environmental data in Korea, such as the National Health Insurance Corporation (NHIC) data and the NIER (National Institute of Environmental Research) air pollution data, need to be collected at more disaggregated administrative units, and be readily available to researchers and professionals in the field. Recent developments in big data analytics can greatly accelerate data-driven research and policy implementation using the large-sized spatiotemporal data on environmental risks and health outcomes [51].

However, there are several limitations inherent in this study. First of all, as this study focuses on the bivariate association between air pollution and allergic diseases, it rules out various environmental and socioeconomic variables or risk factors which may be related to at least one of the allergic diseases. As the level of various social and environmental risk factors tends to be spatially inconsistent and change over time, spatiotemporal variation in the prevalence of allergic diseases can be anticipated accordingly, although this paper looked into spatial and temporal heterogeneity separately, and ignored correlations among air pollutants. Their confounding effects may explain low correlation coefficients, measured by both Pearson’s R and Lee’s L statistics. As a matter of fact, the recent literature attempted to evaluate the impact of various allergens such as pollen count [52] and dust mites [53]. Studies on the effects of climate change on allergic diseases are still lacking in Korea, but the global rise in allergic disease prevalence may be directly or indirectly related to climate change and its aftermath [54,55]. Other limitations relate to the data source involved in this study. The patient count data measured by inpatient and outpatient medical treatment records may have overestimated or underestimated true disease burden due to error or bias in the data reporting process [29]. For instance, true level of exposure should be measured based on actual mobility patterns even beyond their administrative boundary, which cannot be captured by the aggregated patients count data. Moreover, the data fail to incorporate disease symptom severity, which tends to be closely related to air pollutant concentration [56]. Air pollutant concentration data—which are collected from only 25 monitoring stations in Seoul—had to be interpolated to the entire surface of Seoul to match with the patient count data collected at 423 sub-districts. The excessive level of spatial autocorrelation found in air pollutant concentrations could be due to this process of spatial estimation, and the results are subject to this type of spatial interpolation. Neither seasonality nor historical trends of both air pollutants and the disease outcomes were discussed in this study, as it focused more on spatial distribution of annually-aggregated data during the recent five years.

## 5. Conclusions

This study examines bivariate correlation between outdoor air pollutants and the prevalence of allergic diseases, highlighting the limitation of a non-spatial correlation measure, and suggesting an alternative to address spatial autocorrelation. It is also the first to compare two types of bivariate association measures—Lee’s L statistics and Pearson’s correlation coefficient—between ambient air pollution and allergic diseases in Korea at a highly disaggregated scale, emphasizing that not only spatially-clustered phenomenon of the association, but also local instability in their relationships, should be considered in investigating the environmental impact on diseases. Although the association between atopic dermatitis and PM_10_ is still under debate, the findings presented in this study underscore the role of spatial analysis in investigating the contribution of local-level spatiotemporal dynamics of air pollution to trends and distribution of allergic diseases.

## Figures and Tables

**Table 1:** Univariate autocorrelation measures of allergic disease prevalence and air pollutants (global Moran’s coefficient).

		2011	2012	2013	2014	2015
Allergic disease prevalence	Allergic rhinitis (children under 12)	0.260	0.295	0.291	0.284	0.290
	Asthma (children under 12)	0.290	0.339	0.296	0.359	0.346
	Atopic dermatitis (children under 12)	0.329	0.322	0.262	0.254	0.164
Ambient air pollutants	SO_2_	0.844	0.957	0.984	0.982	0.872
	NO_2_	0.833	0.711	0.761	0.876	0.901
	CO	0.796	0.891	0.618	0.957	0.969
	PM_10_	0.961	0.971	0.982	0.838	0.946

Note: All autocorrelation measures are significant at *p* < 0.01.

**Table 2:** Bivariate correlation between air pollutants and allergic disease prevalence: Pearson’s R vs. Lee’s L.

Air Pollutants	Allergic Disease Prevalence	2011		2012		2013		2014		2015	
		Pearson’s R	Lee’s Global L	Pearson’s R	Lee’s Global L	Pearson’s R	Lee’s Global L	Pearson’s R	Lee’s Global L	Pearson’s R	Lee’s Global L
SO_2_	Allergic rhinitis	NS	NS	NS	NS	NS	NS	−0.1	NS	NS	NS
	Asthma	NS	NS	NS	NS	NS	NS	NS	NS	NS	NS
	Atopic dermatitis	NS	NS	NS	NS	NS	NS	−0.151	NS	−0.114	NS
NO_2_	Allergic rhinitis	−0.113	NS	−0.118	NS	NS	NS	NS	NS	NS	NS
	Asthma	NS	NS	−0.11	NS	0.119	0.091	NS	NS	NS	0.054
	Atopic dermatitis	NS	0.063	0.132	0.112	NS	0.055	0.098	0.064	NS	NS
CO	Allergic rhinitis	NS	NS	−0.099	NS	NS	0.039	NS	NS	NS	NS
	Asthma	NS	NS	NS	NS	NS	NS	−0.151	NS	−0.11	NS
	Atopic dermatitis	0.149	0.136	0.16	0.18	NS	NS	NS	0.059	NS	0.077
PM_10_	Allergic rhinitis	−0.114	NS	NS	NS	NS	NS	−0.143	NS	NS	NS
	Asthma	−0.174	NS	−0.114	NS	NS	NS	−0.139	NS	−0.127	NS
	Atopic dermatitis	0.252	0.242	NS	NS	NS	NS	NS	NS	NS	0.045

NS: Not significant at *p* < 0.05.
